# Use of genetic correlations to examine selection bias

**DOI:** 10.1002/gepi.22584

**Published:** 2024-07-30

**Authors:** Chin Yang Shapland, Apostolos Gkatzionis, Gibran Hemani, Kate Tilling

**Affiliations:** ^1^ MRC Integrative Epidemiology Unit University of Bristol Bristol UK; ^2^ Population Health Sciences University of Bristol Bristol UK

**Keywords:** correlation, covariance, selection bias

## Abstract

Observational studies are rarely representative of their target population because there are known and unknown factors that affect an individual's choice to participate (the selection mechanism). Selection can cause bias in a given analysis if the outcome is related to selection (conditional on the other variables in the model). Detecting and adjusting for selection bias in practice typically requires access to data on nonselected individuals. Here, we propose methods to detect selection bias in genetic studies by comparing correlations among genetic variants in the selected sample to those expected under no selection. We examine the use of four hypothesis tests to identify induced associations between genetic variants in the selected sample. We evaluate these approaches in Monte Carlo simulations. Finally, we use these approaches in an applied example using data from the UK Biobank (UKBB). The proposed tests suggested an association between alcohol consumption and selection into UKBB. Hence, UKBB analyses with alcohol consumption as the exposure or outcome may be biased by this selection.

## 1 INTRODUCTION

**Figure 1:**
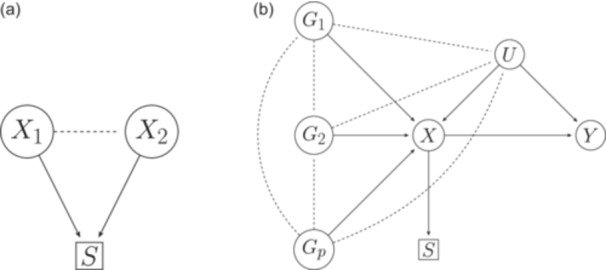
(a) Causal diagram representing collider bias. Two independent variables $X_{1}$ and $X_{2}$ that are both causes of selection (S) will be correlated (shown by the dotted line) when conditioning on S (shown by the box around S). (b) Causal diagrams representing the hypothesized relationship between p genetic instruments, $G_{j}$ (j=1,…,p), an exposure (X), an outcome (Y), a selection variable (S), and all unmeasured variables (U) which confound X and Y. The dotted lines show the correlation between all causes of X induced by conditioning on S.

In statistical inference, it is usually assumed that the analysis sample is representative (i.e., a random subsample) of the target population. This assumption could be violated by participants being nonrandomly selected into the analysis sample. This can occur when measured and unmeasured factors affect initial study enrollment or loss to follow up, or when the analysis is limited to a selected group (e.g., only those with no disease at baseline). The distortion of the parameter estimate between the analysis sample and the true value in the target population is known as “selection bias” (Rothman et al., 2008). Selection bias may also be a form of collider bias (Hernán et al., 2004). Collider bias is induced when conditioning on a common effect (selection, S) of two (or more) variables $X_{1}$ and $X_{2}$, as shown in Figure 1a.

In a complete‐case regression analysis, that is, a regression analysis using only individuals with fully observed data on the exposure, outcome, and other covariates, the selection causes bias in the exposure–outcome regression coefficient estimate if the selection is related to both the exposure and the outcome (or just the outcome for linear regression) (Bartlett et al., 2015; Hughes, Heron, et al., 2019). In Mendelian randomization (MR), a popular method for estimating causal effects using genetic variants as instrumental variables, selection bias may occur if the selection is related to the exposure or outcome (Hughes, Davies, et al., 2019). Therefore, to investigate the likelihood of selection bias in a given analysis, we need to examine whether the exposure or outcome could cause selection. It is possible to examine the association of measured variables with selection if these variables have also been measured in nonselected individuals. If the selection is of participants into a study, then information on nonparticipants is usually nonexistent or minimal, and thus, it is hard to assess which variables are related to selection. However, causes of the exposure (or outcome) that are independent in the target population will become dependent in the selected sample if the exposure (or outcome) causes selection. Thus, correlations between these causes of exposure/outcome observed within the sample would indicate that it is plausible that exposure/outcome caused selection and, therefore, that the exposure–outcome analysis may be biased.

Most studies are prone to nonrandom selection. One example is the UK Biobank (UKBB), which is one of the largest cohort studies with genetic data. UKBB participants have been shown to differ from the UK population in various characteristics (Fry et al., 2017). UKBB is often used for genetic analyses, and in particular MR, and some of these MR analyses have been found to be biased by selection (Munafò et al., 2018). For example, the association between alcohol intake and cardiovascular disease has been found to be underestimated in UKBB (Stamatakis et al., 2021). One aspect of genetic studies that can help with the detection of selection bias is that genetic variants not in linkage disequilibrium (LD) (e.g., those on different chromosomes) should (after quality control) be independent in the target population (Pirastu et al., 2021). If they are found to be correlated in a study sample, one reason for this could be selection bias. More specifically, as shown in Figure 1b, suppose that our objective is to conduct an MR analysis of the effect of an exposure X on an outcome Y, and let $G_{j}$ (j=1,…,p) be independent genetic variants associated with the exposure. Let S represent selection into the study sample: for individual i (i=1,…,n) we let $S_{i}=1$ if that individual is selected into the study and $S_{i}=0$ if they are not. Conditioning on S induces associations between all causes of S; hence, the genetic variants $G_{j}$ will be correlated in the selected sample.

To detect selection bias, one approach is thus to examine associations between variables that should be independent in the target population (e.g., the genetic variants $G_{j}$ in the previous example). One approach would be to examine every pairwise correlation coefficient, but this would require many tests to be conducted, and hence require correction for multiple testing (Larzelere & Mulaik, 1977). Therefore, we have focused on tests based on correlation/covariance matrices. Bartlett (1951), Jennrich (1970), and Steiger (1980) proposed test statistics for comparing correlation matrices. Box's *M* test (Box, 1949) compares covariance matrices. Our aim is to investigate the use of these four hypothesis tests to examine induced associations between genetic variants in a selected sample. We consider two scenarios for identifying selection: “one‐sample” and “two‐sample” analyses. The former examines the evidence for a single sample not being a random subsample of the target population by comparing the observed correlation matrix of genetic variants in that sample to that expected in the target population (the identity matrix). The latter examines the evidence for two different samples not being from the same population. This is particularly relevant in the case of two‐sample MR, which derives causal effect estimates from summary statistics obtained from two separate samples—one provides the *G*–*X* associations and the other provides the *G*–*Y* associations. A key assumption of MR is that both samples come from the same underlying population (Bowden et al., 2017). With individual‐level data on both samples, one could assess which factors are associated with selection into each sample. However, with only summary data from each sample (i.e., the SNP‐exposure or SNP‐outcome coefficients), as is often the case, this cannot be done. Comparison of correlations between genetic variants could instead be used to assess the plausibility of this assumption.

We begin with a motivating example using an MR study from UKBB. Section 2 introduces the different hypothesis testing approaches. Section 3 describes our simulation study, designed using the ADEMP framework (Morris et al., 2019), to evaluate these approaches. Section 4 gives the results of our Monte Carlo simulations. Finally, in Section 5, these approaches are applied to the motivating example to test for the presence of selection bias.

### 1.1 Motivating example: Alcohol consumption in UKBB

A recent MR study showed that alcohol consumption has a causal effect on the risk of having a stroke (Larsson et al., 2020). The instruments used were single nucleotide polymorphisms (SNPs) from a genome‐wide association study (GWAS) on alcohol use (Liu et al., 2019). One of the studies included in this meta‐analysis was UKBB, in which an individual's weekly alcohol intake is only measured if the participant indicated that their alcohol consumption was “more often than once or twice a week.” This exclusion meant that 35% of individuals in the UKBB sample (total *N* = 501,532) did not have weekly alcohol intake measured. We hypothesize that in the selected sample (those with alcohol intake measured), the SNPs associated with alcohol consumption will be correlated with each other. Similarly, Fry et al. (2017) have shown that alcohol consumption is patterned differently in UKBB than in a less selected sample, which would imply that the SNPs associated with alcohol consumption would be correlated with each other in UKBB. This correlation would imply that an MR analysis using alcohol consumption as the exposure or outcome in UKBB could be prone to selection bias.

## 2 METHODS

We first describe the assumed model and define the correlation and covariance matrices for the one‐sample case, and then for the two‐sample case.

In the one‐sample case, suppose that we have p variables of random vector $V={\left(V_{1},\ldots ,V_{p}\right)}^{T}$ following some distribution with mean vector $\mu =\left(\mu_{1},\ldots ,\mu_{p}\right)$ and covariance matrix $𝚺={\left(\sigma_{i,j}\right)}_{p\times p}$. For a sample of n individuals, the sample mean vector is $\bar{V}=\frac{1}{n}\sum_{k=1}^{n}V_{k}$ and the sample covariance matrix $\hat{𝚺}={\left({\hat{\sigma }}_{i,j}\right)}_{p\times p}$ is defined as

(1) $$\hat{𝚺}=\frac{1}{n-1}\sum_{k=1}^{n}\left(V_{k}-\bar{V}\right){\left(V_{k}-\bar{V}\right)}^{T}.$$

The sample correlation matrix is denoted $\hat{R}={\left({\hat{r}}_{i,j}\right)}_{p\times p}$, and is estimated from the sample covariance matrix:

(2) $${\hat{r}}_{i,j}=\frac{{\hat{\sigma }}_{i,j}}{\sqrt{{\hat{\sigma }}_{i,i}{\hat{\sigma }}_{j,j}}},\;\;1\leq i\leq j\leq p.$$

In the two‐sample case, we have two independent random samples, $V_{1},\ldots ,V_{n1}$ and $D_{1},\ldots ,D_{n2}$. The first sample consists of i.i.d. draws from a p‐variate distribution with mean vector $\mu_{1}={\left(\mu_{1,1},\ldots ,\mu_{p,1}\right)}^{T}$ and covariance matrix ${𝚺}_{1}={\left(\sigma_{i,j,1}\right)}_{p\times p}$, and the second sample comes from a distribution with mean vector $\mu_{2}={\left(\mu_{1,2},\ldots ,\mu_{p,2}\right)}^{T}$ and covariance matrix ${𝚺}_{2}={\left(\sigma_{i,j,2}\right)}_{p\times p}$. Define the sample mean vectors by $\bar{V}=\frac{1}{n_{1}}\sum_{k=1}^{n_{1}}V_{k}$ and $\bar{D}=\frac{1}{n_{2}}\sum_{k=1}^{n_{2}}D_{k}$ and the sample covariance matrices ${\hat{𝚺}}_{1}={\left({\hat{\sigma }}_{i,j,1}\right)}_{p\times p}$ and ${\hat{𝚺}}_{2}={\left({\hat{\sigma }}_{i,j,2}\right)}_{p\times p}$ by

(3) $${\hat{𝚺}}_{1}=\frac{1}{n_{1}-1}\sum_{k=1}^{n_{1}}\left(V_{k}-\bar{V}\right){\left(V_{k}-\bar{V}\right)}^{T}\;\;\text{and}\;{\hat{𝚺}}_{2}=\frac{1}{n_{2}-1}\sum_{k=1}^{n_{2}}\left(D_{k}-\bar{D}\right){\left(D_{k}-\bar{D}\right)}^{T}.$$

Let $R_{l}={\left(r_{i,j,l}\right)}_{p\times p}$ be the correlation matrices of sample l, where l=1,2. Then the sample correlation matrices are ${\hat{R}}_{l}={\left({\hat{r}}_{i,j,l}\right)}_{p\times p}$ with

(4) $${\hat{r}}_{i,j,l}=\frac{{\hat{\sigma }}_{i,j,l}}{\sqrt{{\hat{\sigma }}_{i,i,l}{\hat{\sigma }}_{j,j,l}}},\;\;1\leq i\leq j\leq p.$$

In the one‐sample case, if we have external information that the correlation matrix R in the target population is the identity matrix (e.g., if $V={\left(V_{1},\ldots ,V_{p}\right)}^{T}$ are genetic variants, which are expected to be independent after quality control) then our objective is to test the null hypothesis:

I. $H_{0}:R=I$,

where I is the identity matrix. This one‐sample equivalence of the correlation matrix to the identity matrix is formally known as the “Identity Hypothesis.” The equivalent hypothesis for the covariance matrix would be that Σ is a diagonal matrix. We do not test this hypothesis here, as Box's *M* test only assesses whether two covariance matrices are equal, so we would have to make assumptions about the expected variances of the variables $V_{1},\ldots ,V_{p}$ in the full sample to use it. In the two‐sample case, the null hypothesis is

II. $H_{0}:R_{1}=R_{2}$ or $H_{0}:{𝚺}_{1}={𝚺}_{2}$.

In this case, tests can be constructed either for the equality of correlation matrices or for the equality of covariance matrices. Note that in a two‐sample analysis, it is possible that the correlation matrices for the selected and the unselected samples are the same but the covariance matrices differ. For hypothesis (II), we assume that there are no individuals that are included in both samples. As hypothesis testing (I) requires one sample and (II) requires two, we hereafter refer to (I) and (II) as hypothesis tests for one‐sample and two‐sample analyses, respectively.

We are interested in the case where the number of variables (p) is much smaller than the sample size (n). Under this “p<n” assumption, the test statistics used by all following tests are approximately $\chi_{p\left(p-1\right)∕2}^{2}$ distributed (Cai, 2017).

### 2.1 Testing the identity hypothesis with one sample

#### 2.1.1 Bartlett test

Bartlett (1951) proposed the following test statistic:

$$\chi_{B}^{2}=-\left[n-\frac{1}{6}\left(2p+5\right)\right]log|\hat{R}|,$$

which is assumed to be $\chi^{2}$‐distributed with p(p−1)∕2 degrees of freedom.

#### 2.1.2 Jennrich test

Jennrich (1970) defined $\bar{R}=I$, $S=I+\left(\bar{R}{\bar{R}}^{-1}\right)$, c=n, and $Z=\sqrt{c}{\bar{R}}^{-1}\left(\hat{R}-I\right)$. Under the identity hypothesis, the test statistic

$$\chi_{J}^{2}=\frac{1}{2}tr\left(Z^{2}\right)-diag{\left(Z\right)}^{T}S^{-1}diag\left(Z\right)$$

is $\chi^{2}$‐distributed with p(p−1)∕2 degrees of freedom. The first term on the right‐hand side tests the equality of the covariance matrices, and the second term is a correction term for testing the equality of correlation matrices.

#### 2.1.3 Steiger test

Steiger (1980) proposed the following test statistic for the one‐sample case:

$$\chi_{S}^{2}=\left(n-3\right)\sum_{j<i}z_{ij}^{2},\text{where}z_{ij}=\frac{1}{2}log\left[\frac{\left(1+{\hat{r}}_{ij}\right)}{\left(1-{\hat{r}}_{ij}\right)}\right],$$

which again has a $\chi^{2}$ distribution with p(p−1)∕2 degrees of freedom. For small samples, this test statistic performs better than Jennrich's, as Fisher's r‐to‐z transformation ensures the correlation coefficients are normally distributed (Neill & Dunn, 1975).

### 2.2 Testing the equality of correlation/covariance matrices from two samples

#### 2.2.1 Box's *M* test

Box (1949) proposed the *M* test:

$$\chi_{M}^{2}=\left(1-q\right)M,$$

where

$$\begin{array}{ccl} M & = & \sum_{l=1}^{2}v_{l}log|\bar{s}|-\sum_{l=1}^{2}\left(v_{l}log|{\hat{\Sigma }}_{l}|\right),\\ q & = & \frac{2p^{2}+3p-1}{6\left(p+1\right)}\left(\sum_{l=1}^{2}\frac{1}{v_{l}}-\frac{1}{n_{1}+n_{2}-2}\right) \end{array}$$

and $v_{l}=n_{l}-1$, $\bar{s}=\frac{\sum_{l=1}^{2}v_{l}{\hat{\Sigma }}_{l}}{\sum_{l=1}^{2}v_{l}}$. Here, *M* is the test statistic and *q* is a scale factor that ensures the test statistic is asymptotically distributed as $\chi^{2}$ with p(p+1)∕2 degrees of freedom, even with small samples. Note that Box's *M* test can be used to test the equivalence of multiple covariance matrices (by letting the index l take more than two values), but here we have simplified the test statistics to test the equivalence of two covariance matrices.

#### 2.2.2 Jennrich test

Jennrich (1970) described a test for comparing the off‐diagonal of the difference between two correlation matrices ($R_{1}-R_{2}$) against the zero matrix. Let $\bar{R}=\left(n_{1}{\hat{R}}_{1}+n_{2}{\hat{R}}_{2}\right)∕\left(n_{1}+n_{2}\right)$, $S=I+\left(\bar{R}{\bar{R}}^{-1}\right)$, $c=n_{1}n_{2}∕(n_{1}+n_{2})$, and $Z=\sqrt{c}{\bar{R}}^{-1}\left({\hat{R}}_{1}-{\hat{R}}_{2}\right)$. The test statistic proposed by Jennrich is

$$\chi_{J}^{2}=\frac{1}{2}tr\left(Z^{2}\right)-diag{\left(Z\right)}^{T}S^{-1}diag\left(Z\right)$$

with p(p−1)∕2 degrees of freedom. Similar to testing the identity hypothesis for one sample, the first term on the right‐hand side tests the equality of the covariance matrices, and the second term is a correction term for testing the equality of correlation matrices. If the two samples are independently drawn from the same underlying population, then as $n_{1},n_{2}\to \infty$, $\bar{R}$ will tend to the population correlation matrix R and the difference between $R_{1}$ and $R_{2}$ will tend to zero (a matrix with all its elements equal to zero).

#### 2.2.3 Steiger test

For testing the difference between two correlation matrices, the Steiger test statistic (Steiger, 1980) simply becomes

$$\chi_{S}^{2}=\left(N-3\right)\sum_{j<i}X_{ij}^{2},$$

where $X_{ij}=z_{ij1}-z_{ij2}$, $z_{ij1}=\frac{1}{2}log\left[\frac{\left(1+{\hat{r}}_{ij1}\right)}{\left(1-{\hat{r}}_{ij1}\right)}\right]$, $z_{ij2}=\frac{1}{2}log\left[\frac{\left(1+{\hat{r}}_{ij2}\right)}{\left(1-{\hat{r}}_{ij2}\right)}\right]$, and $N=\frac{n_{1}n_{2}}{n_{1}+n_{2}}$, and the test statistic has a $\chi^{2}$ distribution with p(p−1)∕2 degrees of freedom under the null hypothesis of the correlation matrices being equal.

## 3 SIMULATION STUDY PLAN

We conducted a simulation study to compare the performance of the various tests. Here, we describe the design of our simulation study, which follows the ADEMP framework (Morris et al., 2019).

### 3.1 Aims

The simulation study aims to compare the performance (in terms of type‐I error and power) of Bartlett, Jennrich, Steiger, and Box's *M* tests for testing the identity hypothesis in the one‐sample case and the equality of correlation/covariance matrices in the two‐sample case. We compare the performance of the tests across a range of different scenarios, varying the number of genetic variants (SNPs), the sample size (n), and the amount of variance in exposure (X) explained by these SNPs ($R_{GX}^{2}$). The simulations use ranges for $R_{GX}^{2}$ and n that are typically seen in MR studies.

### 3.2 Data‐generating mechanisms

In each simulated data set, we generate genetic data for p independent SNPs ($G_{k}$) and an exposure X for a group of n individuals. The genetic data for SNP $G_{k}$ with minor allele frequency (MAF) $f_{k}$ are generated by first simulating a latent variable $Z_{SNP_{k}}\sim N\left(0,1\right)$, then coding the genotype as 0 if $\Phi \left(Z_{SNP_{k}}\right)<{\left(1-f_{k}\right)}^{2}$, 1 if ${\left(1-f_{k}\right)}^{2}<\Phi \left(Z_{SNP_{k}}\right)<1-f_{k}^{2}$, and 2 otherwise, where Φ() is the cumulative density function of the standard normal distribution. The MAFs for all SNPs are simulated from a uniform distribution, $f_{k}\sim Unif\left(0.1,0.5\right)$.

The exposure X is simulated to be normally distributed. The following describes the relationship between $G_{k}$ and X:

$$X_{i}=\alpha_{0}+\sum_{k=1}^{p}\alpha_{Gk}G_{ki}+\epsilon_{xi},$$

where $\epsilon_{xi}$ are independent random errors distributed as N(0,1), i=1,…,n. The proportion of variance in X explained by the p SNPs is

$$R_{GX}^{2}=\frac{Var\left(\sum_{k}\alpha_{G_{k}}G_{k}\right)}{Var\left(X\right)}=\frac{\sum_{k}\alpha_{G_{k}}^{2}Var\left(G_{k}\right)}{Var\left(X\right)}=\sum_{k}R_{G_{k}X}^{2}.\;$$

$R_{GX}^{2}$ is set equal to 0.45 in most of our simulations, but is varied between 0.05 and 0.45 in some of our simulation scenarios to explore the sensitivity of our results to the value of that parameter (see Table 1).

**Table 1:** Summary of simulation scenarios.

Scenario	p	$R_{GX}^{2}$	n	$\eta_{x}$	t
*One‐sample testing*					
1	50	0.45	2000–10,000	0.988	–
2	10–90	0.45	8000	0.988	–
3	50	0.05–0.45	8000	0.988	–
4	50	0.05	10,000–400,000	0.988	–
5	10–90	0.05	8000	0.988	–
6	50	0.45	8000	0.988, 0.588, 0.188	–
7	50	0.45	8000	–	15, 25, 35, 45
*Two‐sample testing*					
8	50	0.45	5000–20,000	0.988	–
9	10–90	0.45	10,000	0.988	–
10	50	0.05–0.45	10,000	0.988	–
11	50	0.05	10,000–400,000	0.988	–
12	10–90	0.05	10,000	0.988	–
13	50	0.45	10,000	0.988, 0.588, 0.188	–
14	50	0.45	10,000	–	15, 25, 35, 45

Abbreviations: *p*, number of *G*s; *n*, sample size; $R_{GX}^{2}$, proportion of variance in *X* explained by *G*s; *η_x_*, selection pressure on *X*; *t*, quantile of *X* for the selection threshold.

The regression coefficients $\alpha_{G_{k}}$ in the exposure model are specified so that each of the p SNPs in our simulation explains the same proportion of variation in the exposure; $R_{G_{1}X}^{2}=\cdots =R_{G_{p}X}^{2}=R_{GX}^{2}∕p$. Setting $R_{G_{k}X}^{2}=\frac{\alpha_{G_{k}}^{2}Var\left(G_{k}\right)}{Var\left(X\right)}=R_{GX}^{2}∕p$ for SNP k and assuming X is generated to have a variance of 1 yields the following values for the SNP‐exposure effects:

$$\alpha_{G_{k}}^{2}=\frac{R_{GX}^{2}}{p\times Var\left(G_{k}\right)},$$

where $Var\left(G_{k}\right)=2f_{k}\left(1-f_{k}\right)$.

Two types of selection into the study sample are simulated:

- selection completely at random (SCAR);
- selection at random, conditional on *X* (SAR).

Within each simulated data set, 60% of the n individuals are selected (i.e., have exposure data observed). This value was chosen because the proportion of UKBB participants with complete data on a range of commonly used exposures is approximately 60% (Tyrrell et al., 2021) and is similar to the proportion of individuals with recorded weekly alcohol intake data in our motivating example (65%). For the SCAR selection mechanism, 60% of individuals are randomly selected. For SAR, we implemented two different approaches to simulate the selection of participants: a logistic model and a threshold approach. The latter mimics UKBB's data collection in our motivating example: a participant's alcohol frequency was recorded if their weekly alcohol consumption was “more often than once or twice a week.”

The following gives the probability of a participant being selected using a simple logistic model for selection (Hughes, Davies, et al., 2019):

$$P\left(\;\text{participant}\;\;i\;\;\text{selected}\;\right)=expit\left\{\eta_{0}+\eta_{x}X_{i}+V_{i}\right\},$$

where $expit\left\{w\right\}=\frac{exp\left\{w\right\}}{1+exp\left\{w\right\}}$ and V is an independent variable distributed as N(0,1). The values for the parameters $\eta_{0}$ and $\eta_{x}$ were specified to give a mean probability of selection of approximately 0.6, so that 60% of the n individuals have their exposure observed. The inclusion of V allows for another variable, not just X, to influence selection, and introduces more randomness in the selection mechanism. The value of $\eta_{0}$ was set to 0.5, and we used the values 0.988, 0.588, and 0.188 for $\eta_{x}$, which we refer to as strong, moderate, and weak selection pressure on *X*, respectively. Note that X is standardized to ensure that the mean probability of selection is fixed at 0.6 while varying the selection pressure.

For the threshold approach, participants are selected if their value of X is greater than the tth quantile of the distribution of X. The value of the tth quantile is calculated as

$$Q\left(t\right)=\left(1-\gamma \right)x_{\left(j\right)}+\gamma x_{\left(j+1\right)},$$

where $x_{\left(1\right)},\ldots ,x_{\left(n\right)}$ are the exposure values of all n individuals in ascending order, $x_{\left(j\right)}$ is the jth of these values, j is the largest integer not greater than (n+1)t∕100 and γ=(n+1)t∕100−j. We consider four possible values for t: 15, 25, 35, and 45. As the sample sizes of the selected and unselected groups depend on the threshold, and to ensure the sample sizes are the same for every scenario, we first apply the threshold approach to an initial sample of m individuals, where m is greater than n. We then randomly choose (60∗n∕100) individuals from the group with a value of *X* greater than Q(t) and designate this as the selected group. We follow the same procedure for the unselected group, thus choosing (40∗n∕100) individuals from those with a value of *X* less than Q(t).

Table 1 provides a summary of all our simulation scenarios, showing which parameters were varied in each scenario and what values were assigned to those parameters. Each scenario was replicated 1000 times.

We conducted two additional sets of simulations as a form of sensitivity analysis. First, we simulated the variables that cause the exposure to be continuous and normally distributed (instead of genetic variants coded as 0, 1 and 2), to check the robustness of all methods to the distribution of these variables (Supporting Information Note 1.1). Second, we investigated the sensitivity of methods to the selection mechanism. The logistic mechanism we described earlier is known to induce relatively little bias in complete‐case analysis (Gkatzionis et al., 2023). Therefore, we added a quadratic term in *X* to the logistic selection model (Supporting Information Note 1.2).

### 3.3 Methods

In the one‐sample case, the correlation matrix from the selected sample (individuals with exposure observed) is compared with the identity matrix using the following hypothesis tests (as described in Section 2):

1. Bartlett;
2. Jennrich;
3. Steiger.

In the two‐sample case, the correlation/covariance matrix for the unselected sample (individuals without exposure observed) is compared with the correlation/covariance matrix from the selected sample (individuals with exposure observed) using the following hypothesis tests (detailed in Section 2):

1. Box's *M*;
2. Jennrich;
3. Steiger.

### 3.4 Performance measures

The performance of each test is measured by the proportion of simulated data sets in which the test's *p* value was below 0.05. Under SCAR, this represents the test's type‐I error rate and should be equal to 0.05. When selection is based on the exposure (SAR), it represents the test's empirical power to reject the null hypothesis of no correlation (for one‐sample tests) or equal correlations (for two‐sample tests), that is, the power to detect the given selection mechanism.

## 4 RESULTS

### 4.1 Testing the identity hypothesis using one sample

When testing the null hypothesis that the correlation matrix in the selected sample is equal to the identity matrix, Steiger, Jennrich, and Bartlett tests maintained an approximately nominal 5% type I error rate (T1E) under SCAR for all scenarios (Tables 2 and S1). The power of all three methods to detect SAR when the selection mechanism was logistic regression (with only a linear term for the exposure) was low. With this mechanism, the highest power (30%) was observed when a small number of genetic variants (10) explained a large amount of variation in the exposure (45%). However, when the selection mechanism was based on the threshold model (rather than the logistic model), the power of all methods was higher. Power increased with the threshold because the selected group consisted of individuals with values of *X* greater than Q(t) (as described in Section 3.2). As t increased, the selected group contained individuals with higher values of *X*, meaning that the selected sample was more likely to contain individuals with high values of several genetic variants, and less likely to contain individuals with no risk alleles on any genetic variants.

**Table 2:** Proportion of tests with *p* < 0.05 in 1000 simulated data sets when testing the identity hypothesis using one sample.

Test statistics	Bartlett		Jennrich		Steiger	
Miss.	SCAR	SAR	SCAR	SAR	SCAR	SAR
*Scenario 1:* n (p=50 *and* $R_{GX}^{2}=0.45$)						
2000	0.06	0.04	0.07	0.05	0.06	0.05
4000	0.06	0.07	0.06	0.07	0.06	0.07
6000	0.05	0.07	0.06	0.07	0.06	0.07
8000	0.04	0.07	0.04	0.07	0.04	0.07
10,000	0.06	0.08	0.06	0.08	0.06	0.08
*Scenario 2:* p ($R_{GX}^{2}=0.45$ *and* n=8000)						
10	0.04	0.30	0.04	0.29	0.04	0.29
30	0.04	0.12	0.04	0.11	0.04	0.11
50	0.06	0.07	0.07	0.07	0.06	0.07
70	0.05	0.08	0.05	0.08	0.05	0.08
90	0.05	0.06	0.05	0.07	0.05	0.07
*Scenario 3:* $R_{GX}^{2}$ (p=50 *and* n=8000)						
0.05	0.05	0.04	0.05	0.05	0.05	0.05
0.25	0.04	0.07	0.04	0.07	0.04	0.07
0.45	0.05	0.08	0.05	0.07	0.05	0.07
*Scenario 4:* n *with low* $R_{GX}^{2}$ (p=50 *and* $R_{GX}^{2}=0.05$)						
8000	0.05	0.04	0.05	0.05	0.05	0.05
100,000	0.05	0.06	0.05	0.06	0.05	0.06
200,000	0.05	0.06	0.05	0.06	0.05	0.06
400,000	0.06	0.06	0.06	0.06	0.06	0.06
*Scenario 6:* $\eta_{X}$ (p=50, $R_{GX}^{2}=0.05$, *and* n=8000)						
Strong (0.988)	0.05	0.08	0.05	0.07	0.05	0.07
Moderate (0.588)	0.06	0.06	0.06	0.06	0.06	0.06
Weak (0.188)	0.06	0.07	0.06	0.06	0.06	0.06
*Scenario 7:* t (p=50, $R_{GX}^{2}=0.45$, *and* n=8000)						
15	0.04	0.35	0.04	0.30	0.04	0.30
25	0.05	0.68	0.05	0.60	0.05	0.60
35	0.05	0.86	0.06	0.80	0.05	0.80
45	0.06	0.95	0.05	0.91	0.05	0.91

*Note*: The results from scenario 5 are reported in Table S1.

Abbreviations: Miss., Missingness; SAR, selection at random, conditional on *X*; SCAR, selection completely at random; *p*, number of *G*s; *n*, sample size; $R_{GX}^{2}$, proportion of variance in *X* explained by *G*s; *t*, quantile of *X* for selection threshold.

In our simulations with normally distributed variables, the T1E and power of the various methods were very similar to the results for the categorical SNPs (Supporting Information Note 1.1 and Table S3). In our simulations with a quadratic term in the selection model, the power of the one‐sample tests increased, particularly when a small number of genetic variants explained a large proportion of the variance (Table S5).

### 4.2 Testing the equality of correlation/covariance matrices using two samples

When testing the equality of correlation/covariance matrices from two samples under SCAR, all three methods considered had approximately nominal 5% T1E (Tables 3 and S2). The power of the Steiger and Jennrich tests to detect SAR was low in all cases simulated, with the highest power (34%) being for a threshold selection mechanism (Scenario 14). In contrast, Box's *M* test generally showed higher power (>80%), except in Scenarios 10 and 11 where the proportion of variance in X explained by genetic variants was small, and in Scenario 13 where the association between exposure and selection was weak.

**Table 3:** Proportion of tests with *p* < 0.05 in 1000 simulated data sets when testing the equality of correlation/covariance matrices from two samples.

Test statistics	Box's *M*		Jennrich		Steiger	
Miss.	SCAR	SAR	SCAR	SAR	SCAR	SAR
*Scenario 8:* n (p=50 *and* $R_{GX}^{2}=0.45$)						
5000	0.04	0.75	0.07	0.09	0.05	0.05
10,000	0.03	1.00	0.06	0.06	0.05	0.05
15,000	0.04	1.00	0.06	0.05	0.05	0.04
20,000	0.04	1.00	0.06	0.05	0.05	0.04
*Scenario 9:* p ($R_{GX}^{2}=0.45$ *and* n=10,000)						
10	0.04	1.00	0.06	0.06	0.06	0.05
30	0.04	1.00	0.07	0.05	0.06	0.04
50	0.04	1.00	0.07	0.06	0.05	0.05
70	0.02	0.95	0.07	0.07	0.04	0.05
90	0.04	0.86	0.12	0.12	0.05	0.05
*Scenario 10:* $R_{GX}^{2}$ (p=50 *and* n=10,000)						
0.05	0.03	0.13	0.06	0.06	0.04	0.05
0.25	0.04	0.84	0.07	0.06	0.05	0.04
0.45	0.04	0.99	0.07	0.07	0.05	0.05
*Scenario 11:* n *with low* $R_{GX}^{2}$ (p=50 and $R_{GX}^{2}=0.05$)						
10,000	0.03	0.13	0.06	0.06	0.04	0.05
100,000	0.04	1.00	0.05	0.06	0.05	0.05
200,000	0.04	1.00	0.05	0.05	0.04	0.05
400,000	0.04	1.00	0.04	0.05	0.04	0.05
*Scenario 13:* $\eta_{X}$ (p=50, $R_{GX}^{2}=0.05$, *and* n=10,000)						
Strong (0.988)	0.03	0.99	0.06	0.05	0.04	0.04
Moderate (0.588)	0.04	0.46	0.06	0.06	0.05	0.04
Weak (0.188)	0.04	0.05	0.06	0.06	0.04	0.04
*Scenario 14:* t (p=50, $R_{GX}^{2}=0.45$, *and* n=10,000)						
15	0.04	1.00	0.07	0.34	0.06	0.15
25	0.04	1.00	0.07	0.14	0.05	0.08
35	0.04	1.00	0.06	0.07	0.04	0.04
45	0.04	1.00	0.05	0.07	0.04	0.05

*Note*: The results from scenario 12 are reported in Table S2. Miss., Missingness; SAR, selection at random, conditional on *X*; SCAR, selection completely at random; *p*, number of *G*s; *n*, sample size; $R_{GX}^{2}$, proportion of variance in *X* explained by *G*s; *t*, quantile of *X* for selection threshold.

The simulated data sets in Scenario 11 have the low variance explained and large sample size that is typically seen in MR studies. Power did not increase with sample size for either Steiger or Jennrich's tests. Reducing the number of SNPs from 90 to 10 (Scenario 12) did not improve the power for Steiger or Jennrich's tests, but did so for Box's *M* test (Table S2).

One possible reason why Box's *M* test has better power than the Jennrich and Steiger tests is that Box's *M* tests the difference in covariance matrices, thereby comparing variances and covariances between the two samples. When using a logistic selection model, the log‐odds of selection are a linear function of normally distributed covariates if the variances of the covariates in the selected and unselected samples are equal (Chêne & Thompson, 1996). However, if the covariates are not normally distributed, their variance may be different in the selected and unselected samples, even under the logistic selection model. Because Box's *M* test compares covariance matrices, it is more sensitive to this difference in variances than the tests based on correlation matrices. The simulation with normally distributed variables as causes of the exposure gives evidence for this explanation—all tests had nominal T1E but similar (low) power across all scenarios (Supporting Information Note 1.1 and Table S4).

The power of the Box *M*, Jennrich, and Steiger tests increased to 0.99, 0.47, and 0.21, respectively, when the threshold was set at the 15% quantile of *X*. When using $X^{2}$ in the logistic selection model (Supporting Information Note 1.2 and Table S6), Box's *M* and Steiger tests had nominal T1E across all scenarios, while Jennrich's test showed inflated T1E with small sample size and a large number of SNPs. Two‐sample tests were able to detect selection (power above 0.8) unless $R_{GX}^{2}$ was at 0.25 and 0.05, and the total sample size was less than 4000.

## 5 APPLIED DATA EXAMPLE: ALCOHOL CONSUMPTION IN UKBB

Our motivating example of weekly alcohol consumption in UKBB was described in the Introduction. Here, we give details about the selection of instruments for the analysis, as well as quality control, excluding SNPs with low MAF and LD. We then describe how we used the genetic instruments associated with alcohol consumption, body mass index (BMI), and a random set of genetic variants to explore: (1) selection bias in UKBB by using the one‐sample methods to compare the genetic correlation matrix estimated in UKBB to the identity matrix, and (2) selection bias in the analytical sample of those with weekly alcohol consumption measured, by comparing the correlation matrices between those with and without the exposure measured.

### 5.1 Method

A large meta‐analysis of GWAS (N=941,280) identified 99 SNPs associated with weekly alcohol intake (Liu et al., 2019). As negative controls, we randomly selected 99 independent SNPs from across the genome which were present in UKBB (after quality control). As a positive control, we selected 82 previously identified BMI SNPs (Locke et al., 2015). The former (random SNPs) are unlikely to be related to selection either into UKBB or into the analysis sample with weekly alcohol intake measured. For the latter (BMI SNPs), there is evidence that obesity levels in the UKBB are lower than in the general population (Fry et al., 2017), and thus, we expect these SNPs to be correlated in the UKBB sample. We do not expect selection into the sample with alcohol intake measured to depend on BMI; therefore, the correlation matrices between BMI SNPs in those with and without weekly alcohol consumption measured should not differ.

There are a total of 391,872 individuals with genotype data in UKBB after restricting the sample to European, unrelated individuals and imputation accuracy greater than 0.8 (Mitchell et al., 2019). We restricted our analyses to SNPs with MAF between 0.1 and 0.5, SNPs with less than 0.1% missing values, and SNPs that are not in LD (using $r^{2}=$ 0.01 as a threshold, and estimating $r^{2}$ from the 1000 Genomes European data set). After this quality control, we were left with 28 SNPs associated with alcohol consumption, 30 of the randomly selected SNPs, and 27 SNPs associated with BMI. In total, the alcohol intake SNPs explain 1% of the variance in alcohol intake, and the BMI SNPs explain 2% of the variance in BMI. In UKBB with European ancestry, 45.4% of individuals had weekly alcohol intake observed, and 99.7% had their BMI measured. See Tables S7–S9 for phenotypic associations with the three sets of SNPs used. The 1000 Genomes Project was used as a reference data set from which to estimate the LD structure between SNPs, and GWAS associations with alcohol intake were obtained from the TwoSampleMR R package (version 0.5.6).

When testing the identity hypothesis using one sample, the correlation/covariance matrix of each set of SNPs was estimated using data from all European individuals in UKBB and was compared against the identity matrix. This test examines whether the genetic variants for each of the three phenotypes (alcohol consumption, BMI, and random) are plausibly related to selection into UKBB. For the two‐sample hypothesis tests (i.e., testing the equality of the two correlation/covariance matrices), the matrix derived from the selected sample (those with alcohol intake measured) was tested against the matrix from the nonselected sample (those without alcohol intake measured). This test assesses the association of the genetic variants for each of the three phenotypes (alcohol consumption, BMI, and random) with having weekly alcohol intake observed. We know that there is an association between alcohol consumption and having alcohol intake measured, as weekly alcohol intake data were only recorded in UKBB for individuals who drink frequently, therefore the aim of this analysis was to evaluate how well the various methods detect this known selection mechanism.

### 5.2 Results

When testing the identity hypothesis in one‐sample analyses, as shown in Table 4, all tests indicated no evidence against the null hypothesis of no correlation between the randomly selected SNPs in the full UKBB sample. All tests show evidence of correlation between the alcohol intake SNPs, as well as between BMI SNPs, in the full sample. This suggests that alcohol intake and BMI may affect selection into UKBB.

**Table 4:** *p* Value from testing the identity hypothesis in one sample with 391,872 individuals with genotype data in UKBB.

Test	Alcohol intake	Random	BMI
Bartlett	$1.73\times 10^{-312}$	0.193	$6.32\times 10^{-4}$
Jennrich	$1.00\times 10^{-311}$	0.195	$6.06\times 10^{-4}$
Steiger	$4.70\times 10^{-313}$	0.195	$6.06\times 10^{-4}$

Abbreviations: BMI, body mass index; UKBB, UK Biobank.

When testing the equality of correlation/covariance matrices from two samples (Table 5), Box's *M* test showed evidence against the equality of the two covariance matrices for SNPs affecting alcohol consumption (*p* 0.001). Box's *M* test did not show evidence against the equality of covariance matrices between those with and without weekly alcohol intake data for the set of randomly selected SNPs, nor for the set of SNPs associated with BMI (the test's *p* values were 0.598 and 0.649, respectively). The Steiger and Jennrich tests showed no evidence against the equality of correlation matrices between the two samples for any of the three sets of SNPs. This is consistent with our simulations showing that the Jennrich and Steiger tests had low power to differentiate between the “missing completely at random” and “missing at random conditional on X” selection mechanisms, even when the selection was based on the threshold approach.

**Table 5:** *p* Value from two‐sample testing in UKBB, comparing the correlation/covariance matrices between those with and without exposure recorded.

Test	Alcohol intake	Random	BMI
Steiger	0.119	0.888	0.311
Jennrich	0.110	0.886	0.303
Box's *M*	$4.20\times 10^{-18}$	0.598	0.649

Abbreviations: BMI, body mass index; UKBB, UK Biobank.

In conclusion, all the one‐sample tests demonstrated evidence for selection due to alcohol intake into the full UKBB sample. In addition, Box's *M* test was able to confirm that selection into the subsample of individuals with weekly alcohol intake measured was influenced by alcohol consumption. It should be noted that these tests are only able to detect the presence of selection, not to assess the magnitude of any bias. In practice, whether selection causes substantial bias will depend on the specific analysis conducted (Hughes, Heron, et al., 2019).

## 6 DISCUSSION

The Monte Carlo simulation demonstrated that when testing the identity hypothesis using one sample, Steiger, Jennrich, and Bartlett tests have nominal T1E rates when selection is completely at random. However, all three tests had low power across all scenarios examined, except when the selection model was not a simple logistic regression model. When testing the equality of correlation/covariance matrices using two‐sample tests, all three tests we considered had nominal T1E. However, only Box's *M* test had adequate power to detect SAR in most of the scenarios tested. We would not recommend the Jennrich and Steiger tests for testing the equality of correlation matrices from two samples with SNPs (i.e., nonnormal variables), as our simulation study suggests that these tests had limited ability to distinguish SCAR from SAR conditional on X.

Our simulations demonstrated that the selection mechanism and the distribution of variables causing the exposure can influence the performance of the various tests. If the selection model is a standard logistic regression model, then the induced correlation between continuous, normally distributed covariates will be low (Gkatzionis et al., 2023) and their variance is likely to be similar in the selected and unselected samples (Chêne & Thompson, 1996). This could explain the low power of all three tests in this simulation scenario. However, when SNPs are coded as 0, 1 and 2, and selection is based on a logistic model, the variance of the SNPs may differ between the selected and unselected samples (Chêne & Thompson, 1996). This, in turn, means that the covariance matrix of SNPs in the selected sample may differ from that in the unselected sample. In this case, Box's *M* test can detect selection, while the other tests may have lower power (because they focus on correlations and not variances).

One previous Monte Carlo simulation study showed that the power of the Steiger and Bartlett tests increases with sample size (Brown & Forsythe, 1974). Another study found that Bartlett's test is sensitive to nonnormality (Layard, 1973), with an inflated T1E rate for variables that follow a heavy‐tailed distribution. Past studies have also noted that Box's *M* test is robust to nonnormality (Box, 1953; Layard, 1973). Yang and DeGruttola (2012) developed a bootstrap version of Bartlett's test to correct inflated T1E rates in the presence of heavy‐tailed error distributions. However, their Monte Carlo simulation used small sample sizes. Modern GWAS studies typically include large sample sizes of thousands of individuals, and implementing this bootstrap approach in such large studies would come at a substantial computational cost. Within a GWAS setting, Jennrich's test statistics have been used for detecting whether shared risk variants between two traits are the result of “subgroup heterogeneity” or “whole‐group pleiotropy” (Han et al., 2016). Han et al. (2016) improved the low power of Jennrich tests by including allele frequencies and effect sizes as weights. The tests reviewed in our study are designed for a low‐dimensional setting (where the number of variables is smaller than the sample size). For an extensive review of test statistics suitable for high‐dimensional settings, see Zheng et al. (2019).

In our applied example, we identified factors related to selection into both UKBB and the subsample with alcohol intake measured. The design of the questions for UKBB means that only individuals with higher weekly alcohol consumption had their alcohol intake measured. The covariance test correctly identified that SNPs associated with alcohol consumption had a different covariance matrix in the sample with data on alcohol intake than in the sample without. This finding has implications for analyses based on UKBB data that use alcohol intake as the exposure or outcome. In this example, the selection mechanism was known (by the design of the survey), and we used the various tests as proof of principle. In applications where the selection mechanism is unknown, comparing correlation/covariance matrices for genetic variants related to the exposure and outcome between those selected and not selected will help identify whether the exposure or outcome causes selection and, therefore, might lead to selection bias. In our analysis of selection into UKBB, the correlation tests implied that SNPs for both alcohol consumption and BMI were correlated within the UKBB sample. Both alcohol consumption and obesity have been shown to be differently distributed in UKBB participants compared with the general population (Fry et al., 2017), and the correlation tests were able to identify these selection mechanisms. Analyses in UKBB with either BMI or weekly alcohol consumption as the exposure or outcome may be biased by this selection—for a specific analysis, further examination of the other factors involved in the selection mechanism would be required (Hughes, Heron, et al., 2019).

In an applied analysis, the genetic correlation/covariance matrix can be estimated in individuals who have complete genetic data for all SNPs included in the analysis. In practice, some individuals may have missing data for some of the SNPs; this is usually due to poor genotyping quality or technical issues (Pompanon et al., 2005). This missingness is likely to be completely at random, therefore using only individuals with complete data will not cause bias in estimating the correlation/covariance matrix of SNPs. However, such a complete‐case analysis would have reduced sample size and thus lower power. Alternatively, if a few SNPs have missing data for many individuals, these SNPs could be removed from the analysis. Or, as a third option, the values of the missing SNPs could be imputed, based on the values of SNPs in LD.

A key advantage of the tests presented here is that they can be used to detect selection (and thus the likelihood of selection bias) even without data on the unselected sample. This is obviously the case for one‐sample tests, but can also be done for two‐sample tests by comparing genetic correlations in the selected sample to a correlation matrix obtained from a reference (e.g., from the 1000 Genomes Project). In comparison, most methods that aim to detect or adjust for selection bias require access to data from an unselected sample, for example, to model the selection mechanism (Hughes, Heron, et al., 2019). Usually, such methods cannot be implemented without any data for the unselected group. However, in analyses that use genetic data or other variables that are known to be independent in the target population, the tests we have described in this paper can exploit the independence of such variables to explore the selection mechanism using just the observed data.

In practice, an applied researcher working on a specific research question using a particular data set should use knowledge about the pattern of missing data in that data set to identify which selection mechanisms would bias their results. For example, in an MR analysis of alcohol intake on BMI, if selection into the study sample is related to alcohol intake, selection bias can occur (Hughes, Davies, et al., 2019). This could be investigated further by taking SNPs identified as related to the exposure or outcome (here, SNPs related to alcohol intake or BMI) and examining their correlation in the analysis sample. Any observed correlation between these SNPs would indicate that the selection mechanism was caused by those SNPs, and thus, by implication, caused by the exposure/outcome, and thus that a complete‐case analysis may be biased.

Another use of these correlation tests is in two‐sample MR. The two‐sample tests for the equality of correlation/covariance matrices can be used to examine whether the two samples come from the same underlying population. This can be done even if the SNPs included in the two‐sample MR analysis are correlated. Another useful diagnostic for this task would be to compare minor allele frequencies for each SNP between the two samples.

Selection is not the only possible explanation for correlations between genetic variants in a given sample. Assortative mating is another potential explanation. Previous work used genetic correlations to detect assortative mating for genetically predicted traits within a population (Yengo et al., 2018), where under the null hypothesis of random mating, the correlation is zero between alleles in different chromosomes. Assortative mating can be examined using family data; however, GWAS family data are sparse and within‐family analyses usually lack power as they require large numbers of mother–father–offspring trios (Howe et al., 2022). For the two‐sample tests, if assortative mating is the same in two samples being compared (e.g., if they do come from the same population), the correlation matrices from the two samples will not differ. For one‐sample tests, assortative mating and other forms of selection will not be separable as the one‐sample tests assume independence between the SNPs in the underlying population.

Technically, it is possible that SNP–SNP interactions may negate the correlation between SNPs induced by selection. For example, suppose that two SNPs, $G_{1}$ and $G_{2}$, interact in their effect on an exposure X, and X causes selection. The strength of correlation induced between $G_{1}$ and $G_{2}$ will then depend both on the independent effects of each SNP on the exposure and on the strength of their interaction. Sometimes it may happen that the correlations induced by the independent effects and the interaction cancel out, causing the correlation between $G_{1}$ and $G_{2}$ in the selected sample to be approximately zero (Gkatzionis et al., 2023).

The tests described here assess whether the selection is dependent on the variables $G_{1},\ldots ,G_{p}$, and by implication dependent on *X*. However, this does not give information about other variables related to selection, or about the size of bias induced in any specific analysis. Evidence of selection related to *X* (by rejection of the null hypothesis that “independent” genetic variants for *X* are uncorrelated in the selected sample) implies that selection is likely to bias any analysis in which *X* is an outcome (including an MR analysis where *X* is the exposure, as *X* would be the “outcome” in the first‐stage regression) and could bias an analysis in which *X* was an exposure or a confounder. Lack of evidence of selection does not imply that there is no selection bias—the sample size and power of the tests to detect a selection effect should also be considered. By offering a simple test, we hope to encourage future epidemiologists to check their assumptions about selection. Many applied analyses have failed to acknowledge selection bias, for example, selection bias was overlooked in some studies of COVID‐19 disease risk (Griffith et al., 2020) and studies that lead to exacerbating health disparities (Rojas‐Saunero et al., 2024). If selection is detected, the potential for bias should then be explored further, either using comparisons to other (less selected) data sets, sensitivity analyses or expert knowledge. Inverse probability weighting is one possible way to mitigate the potential selection bias—provided that the selection model can be correctly specified (Mansournia & Altman, 2016; Seaman & White, 2013). Other approaches for minimizing selection bias are described elsewhere (Griffith et al., 2020). However, in most applied analyses, the exact mechanism of selection will generally be unknown, making it harder to adjust for selection bias.

In conclusion, we recommend the use of the Steiger, Jennrich, and Bartlett tests as sensitivity analyses to identify the potential for selection bias in studies where data on unselected individuals are not available. For two‐sample studies, we suggest using Box's *M* test to examine whether the two samples come from the same population. These hypothesis tests should be examined alongside other evidence for selection or assortative mating. These tests will be particularly useful when there are no data in the unselected sample (e.g., for UKBB participation).

## CONFLICT OF INTEREST STATEMENT

The authors declare no conflict of interest.

## Supporting information

Supplementary Information

## Data Availability

Data from UK Biobank is available upon request at https://www.ukbiobank.ac.uk/enable-your-research/apply-for-access. Code for the simulation study and analysis is available at https://github.com/CYShapland/CorTest.
