# Supplementary material for: Use of genetic correlations to examine selection bias
Source: Genet Epidemiol. 2024 Jul 30;49(1):e22584. doi: 10.1002/gepi.22584 (PMC11656042; doi:10.1002/gepi.22584)
Supplement: Supplementary file 1 — Supplementary Information [file GEPI-49-0-s001.pdf]

Supplementary material for: Use of genetic correlations to examine selection bias

Chin Yang Shapland, Apostolos Gkatzionis, Gibran Hemani and Kate Tilling

MRC Integrative Epidemiology Unit at the University of Bristol, U.K.

Population Health Sciences, University of Bristol, U.K.

*Correspondence to chinyang.shapland@bristol.ac.uk

1 Supplementary Note

1.1 Continuous variables

The simulation approach is as described in Section 3, but instead of a set of SNPs G coded in terms of allele counts (0/1/2) we assume that the exposure is affected by p continuous variables, $Z_1, \ldots, Z_p$, that are normally distributed with a mean of 0 and a standard deviation of 1.

One-sample tests with continuous variables had nominal T1E (Supplementary Table S3). The correlation induced by the logistic selection model is too small for any of the one-sample tests to detect (Gkatzionis et al., 2023). The power of all one-sample tests improves when the selection mechanism is generated by the threshold approach, and increases with t, but this is because the values of X become more extreme in the selected group.

Two-sample tests lacked power in all scenarios (Table S4), except when X is selected based on the smallest threshold (Scenario 14). The power of Box's M, Jennrich and Steiger tests increases with decreasing t, as the values of X become more extreme in the unselected group but less extreme in the selected group. This also reduces the variance in the selected group, hence Box's M test performs better than the Jennrich and Steiger tests.

In conclusion, we would only recommend the one-sample tests and Box's M test for continuous variables if the selection is based on the threshold of X.

1.2 Quadratic term in X

Although selection mechanisms are often simulated using simple logistic regression models including only first-order terms in the linear predictor, this type of selection model tends to lead to low bias in estimated associations unless the outcome has a strong association with selection. We explored this by including a quadratic term in the selection model.

The simulation approach was the same as described in Section 3, but instead of including the linear term "$\eta_x X$" in the selection model, we instead included the quadratic term "$\eta_x X^2$", i.e., a non-linear relationship between X and the (logit-)probability of being selected. The intercept η_0 in the selection model was set to -0.33 and η_X was set equal to 0.988 to give a mean probability of selection of 0.6 with standard deviation of 0.2.

All of the one-sample tests have nominal T1E (Table S5) in this simulation. Power to detect selection increases with increasing sample size; however it does not reach 0.8 even with a sample size of 10,000 and $R^2_{GX} = 0.45$. The tests' power only reaches 0.8 in simulations with 30 SNPs. When the variance explained is small (R^2_{GX}=0.05), all three methods require larger sample sizes to gain power to detect selection.

In the two-sample case, Box's M and Steiger's tests have nominal T1E, but Jennrich's test had slightly inflated T1E when the sample size is small and the number of Gs is large (Table S6). Power to detect selection increases with increasing sample size and decreasing number of SNPs. The two-sample tests lack power with R^2_{GX} lower than 0.45. When the variance explained is small (R^2_{GX}=0.05), all three tests require larger sample sizes to gain power to detect selection.

In conclusion, one-sample tests have more power to detect differences in correlation if the sample size and variance explained are large with a small number of Gs. Two-sample tests are more powerful than one-sample tests as they do not require large sample sizes and a small number of Gs but they also lack power when the variance explained is small.

References

Gkatzionis, A., Seaman, S. R., Hughes, R. A., & Tilling, K. (2023). Relationship between collider bias and interactions on the log-additive scale. *arXiv preprint arXiv:2308.00568*.

2 Supplementary Tables

Table S1: Proportion of tests with p-value < 0.05 in 1,000 simulated datasets with testing the identity hypothesis using one sample. R^2_{GX} is 0.05 and sample size of 8,000. Miss.: Missingness; p: number of Gs; N: sample size; R^2_{GX}: total variance in X explained by SNPs.

Test Stats.	**Bartlett**		**Jennrich**		**Steiger**	
Miss.	SCAR	SAR	SCAR	SAR	SCAR	SAR
Scenario 5: $\mathbf{p}$ *with low* $\mathbf{R^2_{GX}}$ *(*$R^2_{GX} = 0.05$ *and* $n = 8000$*)*						
10	0.04	0.04	0.04	0.04	0.04	0.04
30	0.04	0.06	0.04	0.06	0.04	0.06
70	0.05	0.05	0.05	0.06	0.05	0.06
90	0.05	0.05	0.05	0.05	0.05	0.05

Table S2: Proportion of tests with p-value < 0.05 in 1,000 simulated datasets when testing the equality of correlation/covariance matrices from two samples. R^2_{GX} is 0.05 and sample size of 10,000. Miss.: Missingness; p: number of Gs; N: sample size; R^2_{GX}: total variance in X explained by SNPs.

Test Stats.	**Box's M**		**Jennrich**		**Steiger**	
Miss.	SCAR	SAR	SCAR	SAR	SCAR	SAR
Scenario 12: $\mathbf{p}$ *with low* $\mathbf{R^2_{GX}}$ *(*$R^2_{GX} = 0.05$ *and* $n = 10000$*)*						
10	0.04	0.66	0.06	0.05	0.06	0.05
30	0.04	0.24	0.07	0.05	0.06	0.04
70	0.02	0.10	0.07	0.07	0.04	0.04
90	0.04	0.08	0.12	0.11	0.05	0.04

Table S3: Proportion of tests with p-value < 0.05 in 1,000 simulated datasets when testing the identity hypothesis using one sample with continuous normally distributed variables. Miss.: Missingness; SCAR: selection completely at random; SAR: selection at random, conditional on X; p: number of Zs; n: sample size; R^2_{ZX}: total variance in X explained by Z; t: quantile of X for selection threshold.

Test Stats.	**Bartlett**		**Jennrich**		**Steiger**	
Miss.	SCAR	SAR	SCAR	SAR	SCAR	SAR
Scenario 1: **n** *(*$p = 50$ *and* $R^2_{ZX} = 0.45$*)*						
2000	0.05	0.06	0.05	0.06	0.05	0.06
4000	0.05	0.07	0.05	0.08	0.05	0.08
6000	0.05	0.07	0.06	0.07	0.05	0.07
8000	0.06	0.08	0.06	0.08	0.06	0.08
10000	0.05	0.09	0.05	0.08	0.05	0.08
Scenario 2: **p** *(*$R^2_{ZX} = 0.45$ *and* $n = 8000$*)*						
10	0.05	0.32	0.05	0.31	0.05	0.31
30	0.06	0.12	0.06	0.11	0.06	0.11
70	0.05	0.06	0.06	0.06	0.06	0.06
90	0.04	0.07	0.04	0.07	0.04	0.07
Scenario 3: $\mathbf{R^2_{ZX}}$ *(*$p = 50$ *and* $n = 8000$*)*						
0.05	0.05	0.04	0.05	0.05	0.05	0.05
0.25	0.05	0.06	0.05	0.06	0.05	0.06
0.45	0.05	0.08	0.05	0.09	0.05	0.09
Scenario 4: **n** *with low* $\mathbf{R^2_{ZX}}$ *(*$p = 50$ *and* $R^2_{ZX} = 0.05$*)*						
8000	0.05	0.04	0.05	0.05	0.05	0.05
100000	0.04	0.06	0.05	0.06	0.05	0.06
200000	0.05	0.06	0.05	0.06	0.05	0.06
400000	0.04	0.06	0.04	0.06	0.04	0.06
Scenario 5: **p** *with low* R^2_{ZX} *(*$R^2_{ZX} = 0.05$ *and* $n = 8000$*)*						
10	0.05	0.05	0.05	0.06	0.05	0.06
30	0.06	0.05	0.06	0.05	0.06	0.05
70	0.05	0.04	0.06	0.04	0.06	0.04
90	0.04	0.05	0.04	0.05	0.04	0.05
Scenario 6: $\eta_{\mathbf{X}}$ *(*$p = 50$, $R^2_{ZX} = 0.45$ *and* $n = 8000$*)*						
0.988	0.05	0.07	0.05	0.06	0.05	0.06
0.588	0.05	0.06	0.05	0.06	0.05	0.06
0.188	0.05	0.05	0.05	0.05	0.05	0.05
Scenario 7: **t** *(*$p = 50$, $R^2_{ZX} = 0.45$ *and* $n = 8000$*)*						
15	0.05	0.41	0.06	0.36	0.06	0.36
25	0.06	0.72	0.06	0.64	0.06	0.64
35	0.05	0.87	0.05	0.80	0.05	0.80
45	0.06	0.94	0.05	0.90	0.05	0.90

Table S4: Proportion of tests with p-value < 0.05 in 1,000 simulated datasets when testing the equality of correlation/covariance matrices from two samples with continuous normally distributed variables. Miss.: Missingness; SCAR: selection completely at random; SAR: selection at random, conditional on X; p: number of Zs; n: sample size; R^2_{ZX}: total variance in X explained by Z.

Test Stats.	**Box's M**		**Jennrich**		**Steiger**	
Miss.	SCAR	SAR	SCAR	SAR	SCAR	SAR
Scenario 8: $\mathbf{n}$ *($p = 50$ and $R^2_{ZX} = 0.45$)*						
5000	0.05	0.04	0.08	0.07	0.05	0.04
10000	0.04	0.05	0.06	0.06	0.04	0.04
15000	0.03	0.04	0.05	0.06	0.04	0.05
20000	0.05	0.04	0.05	0.05	0.05	0.04
Scenario 9: $\mathbf{p}$ *($R^2_{ZX} = 0.45$ and $n = 10000$)*						
10	0.04	0.05	0.04	0.05	0.04	0.05
30	0.05	0.05	0.06	0.06	0.05	0.05
70	0.04	0.04	0.07	0.06	0.04	0.04
90	0.05	0.04	0.11	0.09	0.04	0.05
Scenario 10: $\mathbf{R^2_{ZX}}$ *($p = 50$ and $n = 10000$)*						
0.05	0.05	0.03	0.06	0.05	0.05	0.04
0.25	0.06	0.06	0.07	0.07	0.06	0.05
0.45	0.05	0.04	0.06	0.05	0.04	0.04
Scenario 11: $\mathbf{n}$ *with low* $\mathbf{R^2_{ZX}}$ *($p = 50$ and $R^2_{ZX} = 0.05$)*						
10000	0.05	0.03	0.06	0.05	0.05	0.04
100000	0.06	0.04	0.06	0.04	0.05	0.04
200000	0.06	0.05	0.05	0.05	0.05	0.05
400000	0.04	0.05	0.04	0.05	0.04	0.05
Scenario 12: $\mathbf{p}$ *with low* R^2_{ZX} *($R^2_{ZX} = 0.05$ and $n = 10000$)*						
10	0.04	0.06	0.06	0.06	0.05	0.06
30	0.05	0.06	0.06	0.07	0.05	0.06
70	0.06	0.04	0.09	0.08	0.05	0.04
90	0.04	0.05	0.13	0.13	0.03	0.05
Scenario 13: $\eta_{\mathbf{X}}$ *($p = 50$, $R^2_{ZX} = 0.45$ and $n = 10000$)*						
0.988	0.05	0.04	0.06	0.06	0.05	0.04
0.588	0.06	0.06	0.07	0.07	0.06	0.06
0.188	0.05	0.04	0.06	0.06	0.04	0.04
Scenario 14: $\mathbf{t}$ *($p = 50$, $R^2_{ZX} = 0.45$ and $n = 10000$)*						
15	0.04	0.99	0.05	0.47	0.04	0.21
25	0.06	0.89	0.07	0.23	0.05	0.11
35	0.05	0.35	0.06	0.11	0.05	0.06
45	0.05	0.07	0.07	0.09	0.05	0.05

Table S5: Proportion of tests with p-value < 0.05 in 1,000 simulated datasets when testing the identity hypothesis using one sample with X^2 in the logistic selection model. Miss.: Missingness; SCAR: selection completely at random; SAR: selection at random, conditional on X; p: number of Gs; n: sample size; R^2_{GX}: total variance in X explained by G; t: quantile of X for selection threshold.

Test Stats.	**Bartlett**		**Jennrich**		**Steiger**	
Miss.	SCAR	SAR	SCAR	SAR	SCAR	SAR
Scenario 1: **n** *(*$p = 50$ *and* $R^2_{GX} = 0.45$*)*						
2000	0.06	0.08	0.07	0.10	0.06	0.10
4000	0.06	0.16	0.06	0.18	0.06	0.18
6000	0.05	0.25	0.06	0.29	0.06	0.28
8000	0.04	0.38	0.04	0.42	0.04	0.42
10000	0.06	0.47	0.06	0.53	0.06	0.53
Scenario 2: **p** *(*$R^2_{GX} = 0.45$ *and* $n = 10000$*)*						
10	0.04	1.00	0.04	1.00	0.04	1.00
30	0.04	0.78	0.05	0.84	0.05	0.84
70	0.05	0.32	0.05	0.37	0.05	0.37
90	0.06	0.22	0.06	0.26	0.06	0.25
Scenario 3: $\mathbf{R^2_{GX}}$ *(*$p = 50$ *and* $n = 10000$*)*						
0.05	0.05	0.05	0.06	0.05	0.06	0.05
0.25	0.06	0.11	0.06	0.12	0.06	0.12
0.45	0.04	0.49	0.05	0.55	0.05	0.55
Scenario 4: **n** *with low* $\mathbf{R^2_{GX}}$ *(*$p = 50$ *and* $R^2_{GX} = 0.05$*)*						
100000	0.05	0.09	0.05	0.09	0.05	0.09
200000	0.05	0.11	0.05	0.12	0.05	0.12
400000	0.06	0.20	0.06	0.20	0.06	0.20
Scenario 5: **p** *with low* R^2_{GX} *(*$R^2_{GX} = 0.05$ *and* $n = 10000$*)*						
10	0.04	0.07	0.04	0.07	0.04	0.07
30	0.04	0.05	0.05	0.05	0.05	0.05
70	0.06	0.04	0.06	0.04	0.06	0.04
90	0.05	0.05	0.05	0.05	0.05	0.05

Table S6: Proportion of tests with p-value < 0.05 in 1,000 simulated datasets when testing the equality of correlation/covariance matrices from two samples with X^2 in the logistic selection model. Miss.: Missingness; SCAR: selection completely at random; SAR: selection at random, conditional on X; p: number of Gs; n: sample size; R^2_{GX}: total variance in X explained by G.

Test Stats.	**Box's M**		**Jennrich**		**Steiger**	
Miss.	SCAR	SAR	SCAR	SAR	SCAR	SAR
Scenario 8: **n** *($p = 50$ and $R^2_{GX} = 0.45$)*						
2000	0.04	0.20	0.17	0.43	0.05	0.20
4000	0.03	0.52	0.08	0.64	0.05	0.49
6000	0.03	0.83	0.07	0.87	0.04	0.81
8000	0.04	0.95	0.07	0.96	0.05	0.94
10000	0.04	0.99	0.07	0.99	0.05	0.99
Scenario 9: **p** *($R^2_{GX} = 0.45$ and $n = 10000$)*						
10	0.04	1.00	0.06	1.00	0.06	1.00
30	0.04	1.00	0.07	1.00	0.06	1.00
70	0.02	0.92	0.07	0.94	0.04	0.90
90	0.04	0.75	0.12	0.85	0.05	0.74
Scenario 10: $\mathbf{R^2_{GX}}$ *($p = 50$ and $n = 10000$)*						
0.05	0.03	0.04	0.06	0.07	0.04	0.05
0.25	0.04	0.34	0.07	0.41	0.05	0.36
0.45	0.04	0.99	0.07	0.99	0.05	0.98
Scenario 11: **n** *with low* $\mathbf{R^2_{GX}}$ *($p = 50$ and $R^2_{GX} = 0.05$)*						
100000	0.04	0.10	0.05	0.13	0.05	0.13
200000	0.04	0.24	0.05	0.28	0.04	0.28
400000	0.04	0.61	0.04	0.66	0.04	0.66
Scenario 12: **p** *with low R^2_{GX} ($R^2_{GX} = 0.05$ and $n = 10000$)*						
10	0.04	0.07	0.06	0.09	0.06	0.09
30	0.04	0.05	0.07	0.07	0.06	0.06
70	0.04	0.04	0.09	0.09	0.06	0.05
90	0.04	0.04	0.10	0.10	0.05	0.05

Table S7: Associations between weekly alcohol intake SNPs and weekly alcohol intake in UK Biobank. Results extracted from *TwoSampleMR* R package (v0.5.10).

SNP	Chr	Pos	EA	NEA	Beta	SE	p-val
rs10506274	12	81601464	T	G	0.011	0.003	5.00E-04
rs1104608	16	73912588	C	G	0.017	0.003	1.70E-08
rs1123285	14	57274519	G	C	0.016	0.003	1.40E-06
rs113443718	16	29892184	A	G	0.027	0.003	6.80E-17
rs11739827	5	166803321	T	G	0.015	0.003	4.50E-07
rs11940694	4	39414993	G	A	-0.044	0.003	1.00E-44
rs12088813	1	66407700	C	A	0.009	0.003	1.20E-02
rs1260326	2	27730940	C	T	-0.051	0.003	3.30E-60
rs12795042	11	133658168	C	A	0.012	0.003	1.40E-04
rs12907323	15	86796012	G	A	-0.008	0.003	9.50E-03
rs13250583	8	20949917	T	C	0.012	0.004	8.80E-04
rs13383034	2	45155276	T	C	-0.020	0.003	5.80E-10
rs2165670	4	100286085	A	G	-0.047	0.005	3.30E-21
rs2764771	16	20013793	A	G	-0.026	0.003	9.80E-15
rs281379	19	49214274	A	G	-0.019	0.003	4.10E-10
rs2854334	17	29715500	G	A	-0.016	0.003	5.30E-07
rs28601761	8	126500031	G	C	-0.013	0.003	3.60E-05
rs3748034	4	3446091	T	G	0.025	0.004	1.40E-08
rs378421	16	28754684	A	G	0.034	0.003	1.80E-27
rs3809162	12	54674235	G	A	-0.027	0.003	6.30E-18
rs4842786	12	92170791	A	G	0.012	0.003	1.20E-04
rs500321	13	27124360	T	A	0.008	0.003	2.80E-02
rs55932213	9	108755622	G	A	-0.012	0.004	6.20E-04
rs56030824	11	47397353	A	G	0.022	0.003	9.90E-12
rs56337305	2	225475560	C	T	0.007	0.003	1.90E-02
rs62250685	3	85457240	G	A	0.021	0.003	3.20E-11
rs6460047	7	73042443	C	T	-0.020	0.004	1.30E-07
rs6951574	7	153489744	C	T	-0.017	0.003	2.30E-08
rs7185555	16	69131281	C	G	0.017	0.004	4.10E-05
rs9838144	3	131576287	C	G	0.021	0.004	4.10E-08

Table S8: Associations between randomly selected SNPs and weekly alcohol intake in UK Biobank. Results extracted from *TwoSampleMR* R package (v0.5.10).

SNP	Chr	Pos	EA	NEA	Beta	SE	p-val
rs11773926	8	24433531	G	A	0.009	0.003	0.0087
rs72771839	5	87763957	A	G	0.015	0.004	0.0002
rs9991896	4	6444182	T	G	0.007	0.004	0.0570
rs2144880	20	59285178	T	C	0.003	0.003	0.3500
rs808233	14	58390589	T	C	-0.001	0.003	0.8000
rs10138601	14	34296324	T	C	-0.005	0.004	0.2500
rs12483418	21	15442979	A	G	-0.003	0.004	0.4500
rs378363	9	9020223	C	T	0.000	0.004	0.9700
rs17335397	8	77230117	C	T	0.003	0.003	0.3200
rs2447862	5	52178971	C	T	-0.000	0.004	0.9900
rs2827550	21	23885063	G	A	0.002	0.004	0.5800
rs12983479	19	36291664	A	G	-0.004	0.005	0.3800
rs61969470	13	86443326	G	A	-0.001	0.004	0.9000
rs10905706	10	10313455	G	A	0.005	0.003	0.1100
rs2223859	20	2393386	A	G	-0.004	0.003	0.1700
rs6070194	20	56211410	C	T	0.001	0.004	0.8400
rs56870642	18	1666022	A	G	0.003	0.003	0.2800
rs34471402	9	103262534	C	T	0.001	0.004	0.8600
rs10060818	5	67859256	G	A	0.019	0.005	0.0001
rs1610395	10	61240403	C	T	-0.002	0.003	0.5500
rs10878056	12	63975707	A	G	-0.002	0.003	0.5100
rs45617131	2	223140055	G	A	0.005	0.004	0.2400
rs759183	7	13295262	A	G	0.003	0.004	0.4700
rs820390	17	73747796	T	A	-0.001	0.004	0.7400
rs2834710	21	36350221	T	C	-0.001	0.003	0.8300
rs7559399	2	208911363	T	C	-0.005	0.003	0.1300
rs464047	5	66161048	G	A	0.007	0.003	0.0260
rs11651622	17	29137155	G	A	-0.011	0.005	0.0150

Table S9: Associations between BMI SNPs and BMI in UK Biobank. Results extracted from *TwoSampleMR* R package (v0.5.10).

SNP	Chr	Pos	EA	NEA	Beta	SE	p-val
rs10182181	2	25150296	G	A	0.033	0.002	3.60E-64
rs10938397	4	45182527	G	A	0.029	0.002	6.90E-48
rs10968576	9	28414339	G	A	0.024	0.002	2.50E-30
rs11030104	11	27684517	G	A	-0.038	0.002	1.30E-53
rs1167827	7	75163169	G	A	0.021	0.002	1.80E-25
rs12446632	16	19935389	A	G	-0.031	0.003	3.90E-27
rs12885454	14	29736838	A	C	-0.017	0.002	7.50E-16
rs12940622	17	78615571	A	G	-0.018	0.002	9.60E-19
rs13021737	2	632348	G	A	0.055	0.003	8.30E-99
rs1516725	3	185824004	C	T	0.032	0.003	1.80E-29
rs1558902	16	53803574	A	T	0.073	0.002	4.90E-291
rs16951275	15	68077168	C	T	-0.029	0.002	1.50E-35
rs17405819	8	76806584	C	T	-0.020	0.002	3.70E-21
rs2033529	6	40348653	G	A	0.022	0.002	1.20E-23
rs2033732	8	85079709	C	T	0.011	0.002	2.40E-06
rs2112347	5	75015242	G	T	-0.027	0.002	2.70E-40
rs2121279	2	143043285	T	C	0.011	0.003	2.60E-04
rs2207139	6	50845490	G	A	0.038	0.003	1.00E-47
rs2245368	7	76608143	T	C	-0.022	0.003	9.80E-17
rs2820292	1	201784287	C	A	0.017	0.002	7.80E-18
rs3810291	19	47569003	A	G	0.028	0.002	8.90E-39
rs3888190	16	28889486	A	C	0.027	0.002	1.20E-39
rs543874	1	177889480	G	A	0.049	0.002	2.70E-91
rs6567160	18	57829135	C	T	0.054	0.002	2.30E-118
rs7138803	12	50247468	A	G	0.029	0.002	9.30E-46
rs9400239	6	108977663	C	T	0.016	0.002	6.00E-14
rs9925964	16	31129895	G	A	-0.022	0.002	9.60E-27
